# Robotic posterior radical antegrade modular pancreatosplenectomy for left-sided pancreatic cancer using the ligament of Treitz first approach: A case report and technical note

**DOI:** 10.1016/j.ijscr.2025.111782

**Published:** 2025-08-06

**Authors:** Kosei Takagi, Tomokazu Fuji, Kazuya Yasui, Kazuyuki Matsumoto, Toshiyoshi Fujiwara

**Affiliations:** a Department of Gastroenterological Surgery, Okayama University Graduate School of Medicine, Dentistry, and Pharmaceutical Sciences, Okayama, Japan; b Department of Gastroenterology, Okayama University Hospital, Okayama, Japan

**Keywords:** Radical antegrade modular pancreatosplenectomy, Distal pancreatectomy, Robotic surgery, Ligament of Treitz, Surgical approach

## Abstract

**Introduction:**

Radical antegrade modular pancreatosplenectomy (RAMPS) is the standardized open surgical technique for treating left-sided pancreatic cancer. However, studies reporting the surgical approaches for robotic RAMPS are limited. Here, we present a robotic posterior RAMPS using the ligament of Treitz first approach.

**Presentation of case:**

A 46-year-old male patient with initially unresectable pancreatic body cancer underwent robotic posterior RAMPS as a conversion surgery after 1-year of chemotherapy with modified FOLFIRINOX.

**Discussion:**

Following evaluation of resectability, the ligament of Treitz first approach was applied. The transverse colon was lifted cranially, and the left renal vein was exposed after dissection around the ligament of Treitz. The left adrenal vein was divided, and the left adrenal gland was resected with special caution to avoid injury to the left renal artery. Retroperitoneal dissection was performed with lymphadenectomy around the superior mesenteric and celiac arteries using the ligament of Treitz first approach. After repositioning the transverse colon, the gastrocolic and gastrosplenic ligaments were dissected. Following the division of the pancreas and splenic vessels, the retroperitoneal dissection line was connected with those of the ligament of Treitz first approach. The operative time was 303 min, and the estimated blood loss was 150 mL.

**Conclusion:**

The ligament of Treitz first approach may be an option for robotic RAMPS for left-sided pancreatic cancer. Surgeons should select the best approach for performing robotic RAMPS.

## Highlights

• Radical antegrade modular pancreatosplenectomy (RAMPS) is the standardized technique for left-sided pancreatic cancer.
• Few studies have reported the surgical approaches for robotic RAMPS.
• This video demonstrated a robotic posterior RAMPS using the ligament of Treitz first approach.
• The ligament of Treitz first approach may be an option for robotic RAMPS for left-sided pancreatic cancer.

## 1 Introduction

Radical antegrade modular pancreatosplenectomy (RAMPS) has been developed as the standardized open surgical technique for left-sided pancreatic cancer that achieves tumor-free margins and radical lymphadenectomy [1]. Although the surgical and oncological feasibility of minimally invasive RAMPS has been reported [2,3], the adoption of RAMPS technique in robotic surgery is still limited due to its technical difficulty and lack of evidence [4,5]. Moreover, robot-specific surgical approaches should be considered to safely perform robotic RAMPS.

In this report, we present a case of a robotic posterior RAMPS using the ligament of Treitz first approach, focusing on surgical techniques. The work has been reported in line with the SCARE criteria [6].

## 2 Case presentation

A 46-year-old male patient with initially unresectable pancreatic body cancer was treated with modified FOLFIRINOX. Following 1-year of chemotherapy, the carbohydrate antigen 19-9 (CA 19-9) level decreased remarkably from 9173 U/mL to normal (30 U/mL). The radiological assessment revealed good local control. Therefore, the patient underwent a robotic posterior RAMPS as a conversion surgery at our institution. The patient's body mass index was 28 kg/m^2^ preoperatively. The operating room should be prepared for open surgery in case of an emergency.

### 2.1 Indication

The surgical indications for robotic RAMPS include tumors diagnosed as stage T1–3 with no distant metastases in accordance with the AJCC TNM staging system [4]. Tumors requiring vascular resection such as the celiac axis, superior mesenteric vein (SMV), and common hepatic artery (CHA), may be contraindicated for robotic surgery.

### 2.2 Positioning

The patient was placed in the supine position with a patient-side surgeon between the legs. Tilt the operating table 7° in reverse Trendelenburg position and 7° to the right.

### 2.3 Surgical technique of the ligament of Treitz first approach in robotic posterior RAMPS

#### 2.3.1 Step 1 evaluation of resectability

**Fig. 1:**
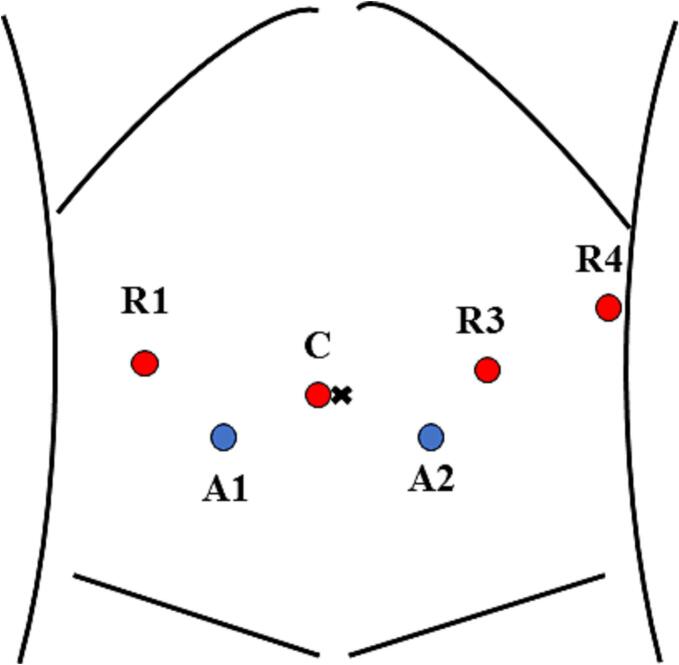
Trocar placement using four robotic trocars at the umbilical level and two trocars for an assistant. R1, robotic arm 1 (Fenestrated Bipolar Forceps); R3, robotic arm 3 (Maryland Bipolar Forceps); R4, robotic arm 4 (Cadiere Forceps); C, camera; A1, 5-mm trocar for an assistant; and A2, 12-mm trocar for an assistant.

Staging laparoscopy was performed to confirm no liver metastases or peritoneal dissemination. Furthermore, the intraoperative peritoneal cytological investigation was negative. Robot docking using a daVinci Xi system (Intuitive Surgical, Sunnyvale, CA, USA) was performed from the right side of the patient. Trocar placement is shown in Fig. 1.

#### 2.3.2 Step 2 the ligament of Treitz first approach

The transverse colon was lifted cranially to dissect the ligament of Treitz. Following the division of the inferior mesenteric vein, the retroperitoneum was dissected to identify the left renal vein (LRV), an important anatomical landmark. The LRV was exposed widely, and the origin of the superior mesenteric artery (SMA) was dissected. By dissecting the ligament of Treitz, the LRV can be easily identified [7]. Moreover, the origin of the SMA can be identified above the LRV.

**Fig. 2:**
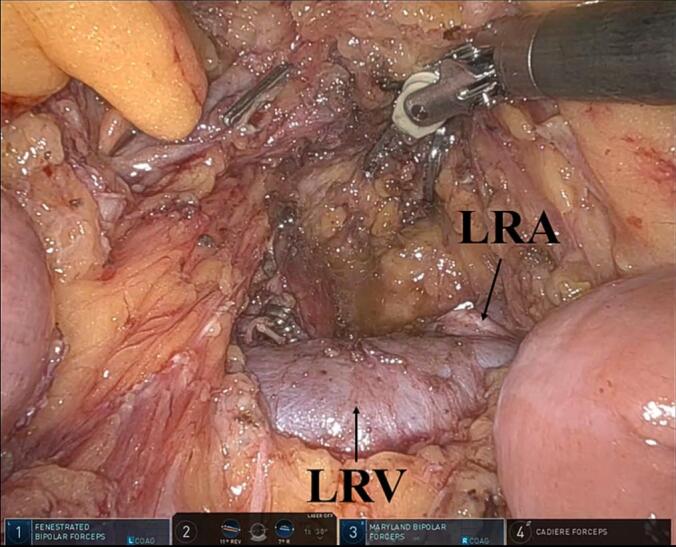
Using the ligament of Treitz first approach, the left renal vein and artery are easily identified. Moreover, the adrenal gland can be resected for posterior radical antegrade modular pancreatosplenectomy. LRA, left renal artery; and LRV, left renal vein.

**Fig. 3:**
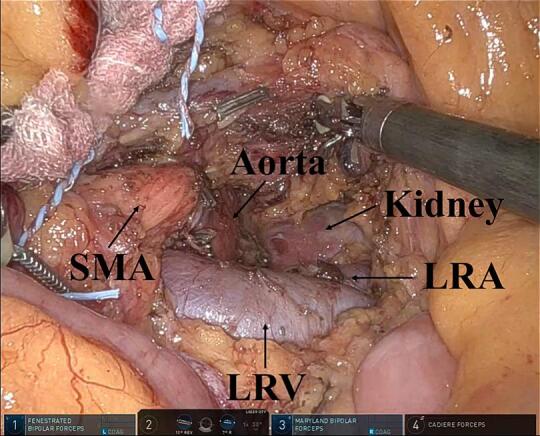
Retroperitoneal dissection and lymphadenectomy around the superior mesenteric artery using the ligament of Treitz first approach. LRA, left renal artery; LRV, left renal vein; and SMA, superior mesenteric artery.

For posterior RAMPS, the left adrenal gland was resected after dividing the left adrenal vein (Fig. 2). The retroperitoneal tissue can be dissected by exposing the left side of the aorta, crus of the diaphragm, and left kidney. Retroperitoneal dissection and lymphadenectomy around the SMA can be performed using the ligament of Treitz first approach (Fig. 3).

#### 2.3.3 Step 3 the gastrocolic ligament division

After repositioning the transverse colon, the gastrocolic ligament was divided extensively. The gastrosplenic ligament can also be dissected at this step.

#### 2.3.4 Step 4 the pancreas and splenic vessels divisions

The superior border of the pancreas was dissected to perform lymphadenectomy around the CHA. After tunneling the pancreas on the SMV, the pancreas was transected on the SMV using a stapler with the progressive stepwise compression technique [8]. Thereafter, the splenic vessels were divided. Surgical approaches to the splenic vessels should be decided depending on their anatomy [9].

#### 2.3.5 Step 5 retroperitoneal dissection

**Fig. 4:**
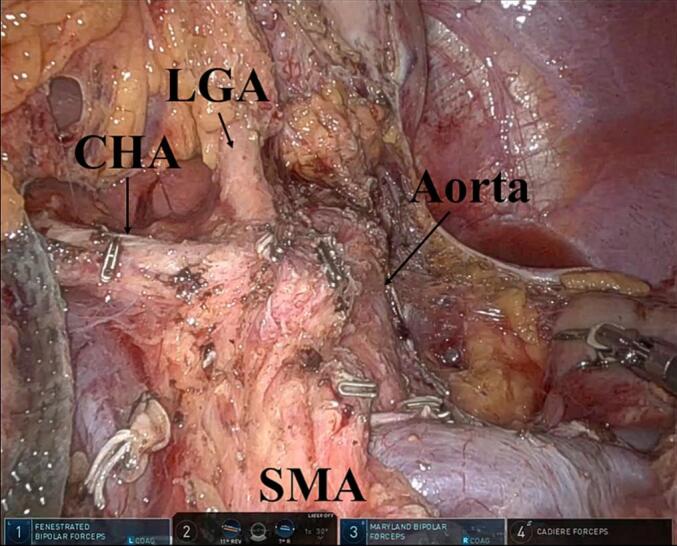
Overview after resection using posterior radical antegrade modular pancreatosplenectomy. CHA, common hepatic artery; LGA, left gastric artery; and SMA, superior mesenteric artery.

After dissecting the anterior plane of the SMA and the left side of the celiac axis, the dissection line was connected with those of the ligament of Treitz first approach. During posterior RAMPS, retroperitoneal dissection proceeded behind the adrenal gland and the anterior Gerota's fascia by exposing the left kidney (level 3) [4]. In contrast, the dissection line should be behind the anterior Gerota's fascia, but in front of the adrenal grand (level 2) for anterior RAMPS [4]. Following the mobilization around the spleen, the specimen was resected (Fig. 4). The specimen was extracted through the Pfannenstiel incision. A drain was placed at the pancreatic stump from the left side of the patient.

### 2.4 Outcomes

The operative time was 303 min, and the estimated blood loss was 150 mL. The patient developed chyle leakage postoperatively but improved with conservative treatment. The postoperative hospital stays were 12 days in this case. The final histopathological examination revealed a well-differentiated invasive ductal carcinoma (T1N0M0 according to the AJCC TNM staging system) with tumor-free margins. The pathological response to chemotherapy was evaluated as grade III (>90 % of tumor cells destroyed) according to the Evans grading system. The patient was alive with stable disease 20 months after surgery.

## 3 Discussion

To the best of our knowledge, this is the first report on robotic posterior RAMPS using the ligament of Treitz first approach. This unique surgical approach allows initial determination of the posterior margin of the pancreas. Although we previously reported robotic RAMPS using the supracolic anterior SMA approach [4], this approach could be more difficult, especially in patients with obesity or those with locally advanced tumors. Therefore, the ligament of Treitz first approach can be an option for patients with such complications.

Using the ligament of Treitz first approach, the LRV can be easily identified during the early phase (step 2) [7]. Subsequently, the left adrenal vein, adrenal gland, SMA, and celiac axis, which are important anatomical landmarks for robotic posterior RAMPS, can be dissected with a good view. Early identification of these important anatomical structures could help to perform retroperitoneal dissection at a late phase after repositioning the transverse colon (step 5). However, careful attention was required not to damage the jejunum during dissecting the ligament of Treitz. The retroperitoneal dissection level should be determined based on the extent of tumor invasion. In this case, tumor invasion into the left adrenal gland was suggested. Therefore, level 3 dissection behind the anterior Gerota's fascia and the adrenal gland was performed. Accordingly, retroperitoneal dissection and lymphadenectomy can be safely performed using the ligament in Treitz's first approach.

## 4 Conclusions

Robotic posterior RAMPS using the ligament of Treitz first approach is safe and feasible for obtaining tumor-free margins and performing radical lymphadenectomy in selected left-sided pancreatic cancers.

## Author contribution

Kosei Takagi: Conceptualization, Patient management, Data curation, Writing- Original draft preparation.

Tomokazu Fuji: Patient management.

Kazuya Yasui: Patient management.

Kazuyuki Matsumoto: Patient management.

Toshiyoshi Fujiwara: Supervision, Writing- Reviewing and Editing.

## Consent

Written informed consent was obtained from the patient for publication and any accompanying images. A copy of the written consent is available for review by the Editor-in-Chief of this journal on request.

## Ethical approval

We certify that this kind of manuscript does not require ethical approval (exemption) by the Ethical Committee of our institution.

## Guarantor

Kosei Takagi.

## Research registration number

Not applicable.

## Funding

There are no sources of funding.

## Conflict of interest statement

The authors have no conflicts of interest to declare.
